# Pituitary stalk interruption syndrome and liver cirrhosis associated with diabetes and an inactivating *KCNJ11* gene mutation: a case report and literature review

**DOI:** 10.3389/fendo.2023.1297146

**Published:** 2023-12-13

**Authors:** Zhaoxiang Liu, Wenhui Zhao, Chenxiang Cao, Yanlei Wang, Luqi Xiao, Xiaojing Wang, Chenxi Jin, Jianzhong Xiao

**Affiliations:** Department of Endocrinology, Beijing Tsinghua Changgung Hospital, School of Clinical Medicine, Tsinghua University, Beijing, China

**Keywords:** pituitary stalk interruption syndrome, liver cirrhosis, KCNJ11, diabetes mellitus, case report

## Abstract

**Background:**

Pituitary stalk interruption syndrome (PSIS) is a congenital disease commonly found in patients with combined pituitary hormone deficiency (CPHD). Most PSIS patients manifest growth retardation and delayed puberty. We report a rare case of PSIS with tall stature, liver cirrhosis and diabetes, possibly caused by an inactivating *KCNJ11* gene mutation.

**Case presentation:**

A 37-year-old female patient initially presented with liver cirrhosis and diabetes, without any secondary sexual characteristics. Endocrine investigation indicated CPHD. Small anterior pituitary, invisible pituitary stalk and no eutopic posterior lobe hypersignal in the sella turcica viewed in magnetic resonance imaging (MRI) confirmed the diagnosis of PSIS. Despite receiving no growth hormone or sex hormone therapy, she reached a final height of 186 cm. Liver histopathology revealed nonalcoholic fatty cirrhosis. Genetic testing identified a heterozygous p.Arg301Cys mutation in the *KCNJ11* gene.

**Conclusion:**

This is a rare case of PSIS with liver cirrhosis and diabetes associated with an inactivating *KCNJ11* gene mutation. It’s supposed that early hyperinsulinism caused by the *KCNJ11* gene mutation, as well as delayed epiphyseal closure due to estrogen deficiency, contributed to the patient’s exceptionally tall stature. Untreated growth hormone deficiency (GHD) resulted in increased visceral fat, leading to nonalcoholic fatty liver disease (NAFLD) and cirrhosis. The decline in β cell function with age, combined with NAFLD, may have played a role in the development of diabetes.

## Introduction

Pituitary stalk interruption syndrome (PSIS) is a rare congenital disorder characterized by varying degrees of pituitary hormone deficiency. The diagnosis of PSIS is confirmed through magnetic resonance imaging (MRI), which reveals the absence of a visible pituitary stalk and ectopic posterior lobe hypersignal in the sella turcica. PSIS patients with growth hormone deficiency (GHD) are able to receive GH replacement therapy during childhood to achieve a normal final adult height (1). In this case report, we present a patient with PSIS who achieved a final height of 186 cm without receiving any GH or sex hormone therapy. Additionally, she was diagnosed with diabetes mellitus and liver cirrhosis. Genetic screening identified an inactivating KCNJ11 mutation, which is believed to cause congenital hyperinsulinism (CHI).

## Case presentation

A 37-year-old female patient presented to our Emergency Department with hematemesis. She had been diagnosed with cirrhosis for two years with an abdominal ultrasonography. Notably, the patient had a remarkable height of 186 cm but did not exhibit any signs of secondary sexual characteristics. After stabilizing her cirrhosis, she was transferred to the Endocrinology Department for further investigation.

The patient was born in a suburban hospital with abnormal medical conditions. There were no detailed birth records on adverse perinatal events. Her mother recalled that she was born at full-term with a birth weight of 3.2 kg (50 centile, 0SD) following a breech delivery (birth length was unknown). There was no definitive record of hypoglycemia in the neonatal period. Her parents were not consanguineous and her father and mother had heights of 176 cm and 150 cm, respectively. The mid-parental height was 156.5 cm (23 centile, -0.8SD). The patient experienced a growth rate of 3-5 cm per year until reaching a height of 186 cm in her twenties. Surprisingly, she had never received any exogenous growth hormone replacement therapy. Moreover, she had no breast development, menarche, pubic hair, or axillary hair. Although prescribed oral estrogen and progesterone for amenorrhea, she exhibited poor compliance with the treatment. The patient had a limited level of learning ability and had only completed elementary school. According to her mother’s recollection, the patient was frequently ill during childhood, but there was no definitive history of hypoglycemia or seizures.

At the age of 24, the patient was diagnosed with diabetes mellitus and initiated metformin and acarbose treatment without regular glucose monitoring. Family history revealed that her parents and several maternal family members had diabetes. Her mother experienced an uneventful pregnancy at the age of 26 and gave birth to the patient, although the blood glucose level during pregnancy was unknown. Her mother was diagnosed with diabetes at the age of 52, while her father was diagnosed at 55. Other maternal family members developed diabetes after the age of 50 and were managed with oral anti-diabetic medications. None of them reported a definite history of childhood hypoglycemia or seizures.

The proband had a height of 186 cm (-> 99 centile, +4.7SD) and weighed 70.3 kg (>97 centile, +2.2SD), with a body mass index (BMI) of 20.32 kg/m^2^ (50 centile, 0SD). Her leg length measured 100 cm, while her arm span was 183 cm. Blood pressure was measured at 126/72 mm Hg, and the heart rate was 76 beats/min. The patient had a Tanner breast I and pubic hair I rating in terms of sexual maturity. Although she had large hands and feet, there were no obvious signs of acromegaly. Furthermore, no abnormalities were detected in the heart and lungs. There were no marfanoid features namely joint hypermobility, arachnodactyly and scoliosis, etc.

### Laboratory investigations

Routine hematology evaluation (**Table 1**) revealed that the patient was anaemic, leucopaenic, and thrombocytopaenic. Liver function tests showed elevated levels of serum alanine aminotransferase, aspartate aminotransferase, and glutamyltransferase.

**Table 1:** Routine hematology test and hormonal measurements at presentation.

	Value	Normal range for age
White blood cells (× 10^9^/L)	2.1	3.5-9.5
Red blood cells (× 10^12^/L)	3.6	3.8-5.1
Hemoglobin (g/L)	106	115-150
Platelets (× 10^9^/L)	61	125-350
Alanine aminotransferase (IU/L)	276	7-40
Aspartate aminotransferase (IU/L)	460	13-35
Glutamyltransferase (IU/L)	57	7-45
Cortisol (nmol/L)	162	166-507
ACTH (ng/L)	3	7-63
LH (IU/L)	<0.07	1.9-12.5 (follicular phase)
FSH (IU/L)	0.32	2.5-10.2 (follicular phase)
Peak LH stimulated by GnRHa (IU/L)	<0.07	>18
Peak FSH stimulated by GnRHa (IU/L)	0.42	
Prolactin (mIU/L)	204	59-619
Estradiol (pg/milliL)	15.17	13.62-166.21 (follicular phase)
Progesterone (ug/L)	<0.21	0-1.4 (follicular phase)
Testosterone (nmol/L)	<0.35	0.31-1.66
GH (ng/mL)	0.06	0.126-9.88
Peak GH stimulated by insulin (ng/mL)	0.19	>10
IGF-1 (ng/mL)	25	96-284
TSH (mIU/L)	11.08	0.38-4.34
FT4 (pmol/L)	6.79	10.45-24.38
FT3 (pmol/L)	2.11	2.77-6.31
Thyroid peroxidase antibody (KU/L)	48.7	< 60
Thyroglobulin antibody (KU/L)	84.0	< 60
17-OHP (pg/mL)	<100	<800 (follicular phase)
DHEA-S (pg/mL)	0.08×10^6^	0.45-2.95×10^6^
HbA1c (%)	10	4-6
Blood glucose (insulin release test, 0min, mmol/L)	6.2	3.9-6.1
Blood glucose (insulin release test, 60min, mmol/L)	9.59	
Blood glucose (insulin release test, 120min, mmol/L)	11.72	
Insulin level (insulin release test, 0min, mIU/L)	7.96	
Insulin level (insulin release test, 60min, mIU/L)	17.37	
Insulin level (insulin release test, 120min, mIU/L)	30.56	
C peptide (insulin release test, 0min, ng/mL)	1.38	
C peptide (insulin release test, 60min, ng/mL)	2.27	
C peptide (insulin release test, 120min, ng/mL)	3.41	
TC (mmol/l)	3.42	< 5.2
TG (mmol/l)	1.03	< 1.7
HDL-c (mmol/l)	0.82	> 1.0
LDL-c (mmol/l)	2.28	< 3.4

HbA1c, glycosylated hemoglobin; ACTH, adrenocorticotropic hormone; LH, luteinizing hormone; FSH, follicle-stimulating hormone; GnRHa, gonadotropin-releasing hormone analogue; IGF-1, Insulin-like growth factor 1; TSH, thyroid stimulating hormone; FT4, free thyroxine; FT3, free triiodothyronine; 17-OHP, 17 hydroxyprogesterone; DHEA-S, dehydroepiandrosterone sulfate; TC, total cholesterol; TG, triglyceride; HDL-c, high-density lipoprotein cholesterol; LDL-c, low-density lipoprotein cholesterol.

**Figure 1:**
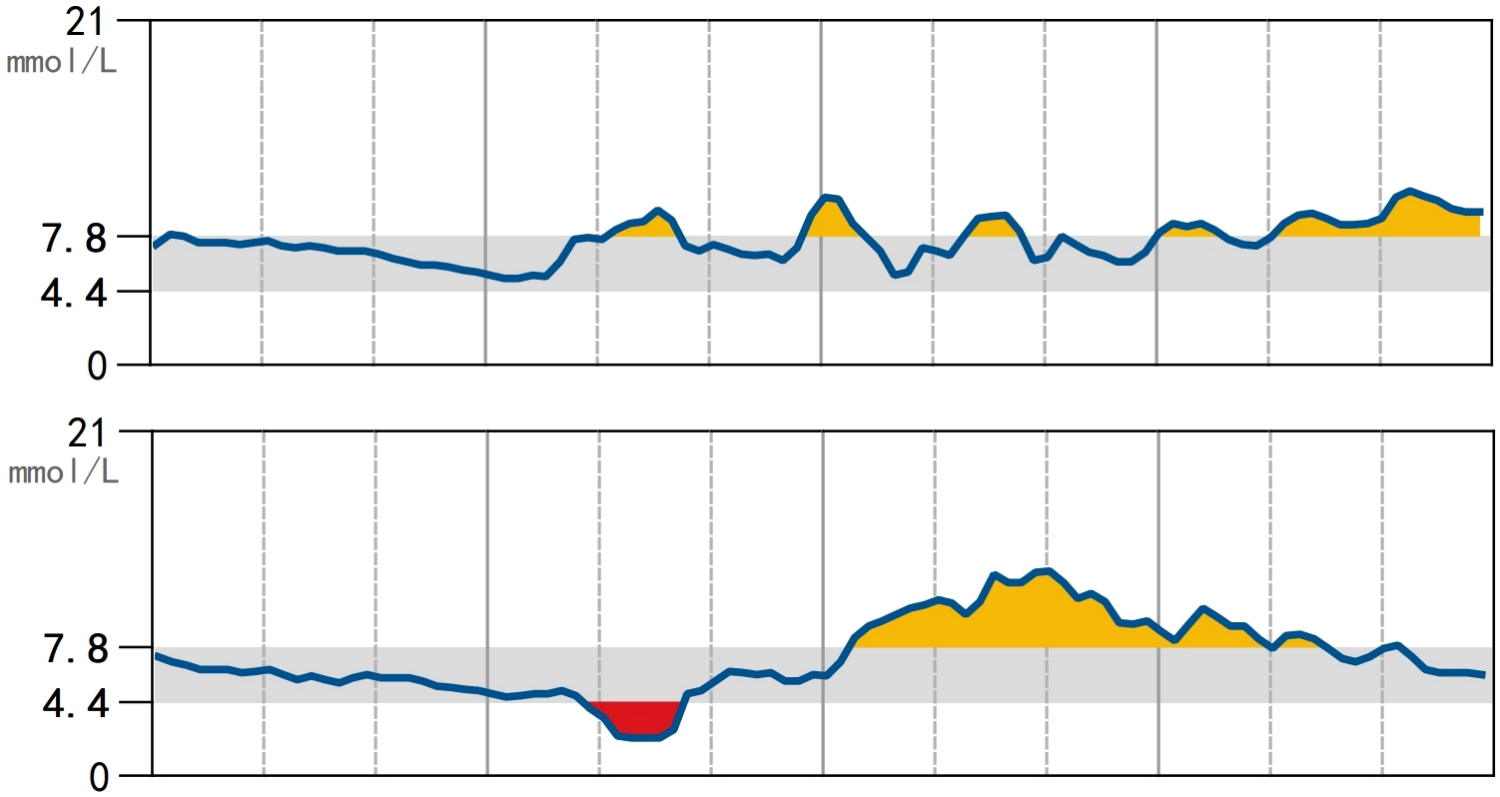
Continuous subcutaneous glucose monitoring using the FreeStyle CGMS Sensor. Blood glucose was measured every minute and recorded every 15 minutes. During a span of 14 days (2 days are shown), hyperglycemia and euglycemia were observed most of the time with incidental hypoglycemia.

Endocrine evaluation (**Table 1**) indicated undetectable estrogen levels with low serum gonadotropin, which did not respond to gonadotropin-releasing hormone analogue (GnRHa) stimulation, suggesting gonadotrophin deficiency. Free thyroxine (FT4) levels were low with slightly elevated thyroid-stimulating hormone (TSH). Serum prolactin levels were within the normal range. Cortisol and adrenocorticotropic hormone (ACTH) levels at 8 am were lower than normal. Blunted response of GH to insulin tolerance test (ITT) indicated GHD. Insulin-like growth factor 1 (IGF-1) levels were also decreased (25 ng/mL). The patient had a female karyotype (46XX). Diabetes-related evaluation showed elevated glycosylated hemoglobin (HbA1c) levels and negative pancreatic islet autoantibodies. The insulin releasing test indicated impaired glucose tolerance anddelayed insulin secretion without absolute insulin deficiency (**Table 1**). Continuous glucose monitoring during hospitalization showed hyperglycemia most of the time, with occasional hypoglycemic events (**Figure 1**).

### Radiology and histopathology

**Figure 2:**
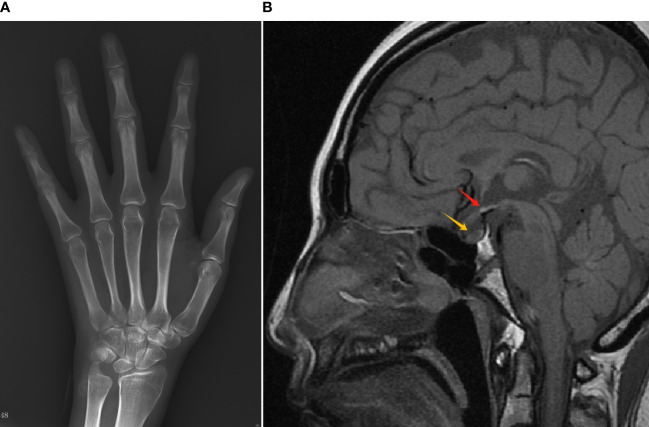
Radiology results of the patient. **(A)** X-ray of the left hand/wrist showed epiphyseal closure. **(B)** MRI of the sella turcica showed a small anterior pituitary remnant (yellow arrow), invisible pituitary stalk, and an ectopic neurohypophysis (red arrow).

X-ray of the left hand and wrist revealed closed epiphysis (**Figure 2A**). MRI showed small anterior pituitary, ectopic neurohypophysis and missing pituitary stalk (**Figure 2B**). Abdominal CT scan revealed signs of portal hypertension, liver cirrhosis, and a small uterus. Echocardiography showed normal left ventricle function. Thyroid ultrasound showed inhomogeneous parenchyma. Carotid artery and lower extremity artery ultrasound results were normal. The results of the ophthalmic evaluation indicated normal vision and fundus, without any mid-line abnormalities.

Ultrasound elastography of liver showed that the controlled attenuation parameter was 265 decibels per meter (dB/m, normal value was < 240). Liver histopathology indicated nonalcoholic fatty cirrhosis.

### Genetic testing

**Figure 3:**
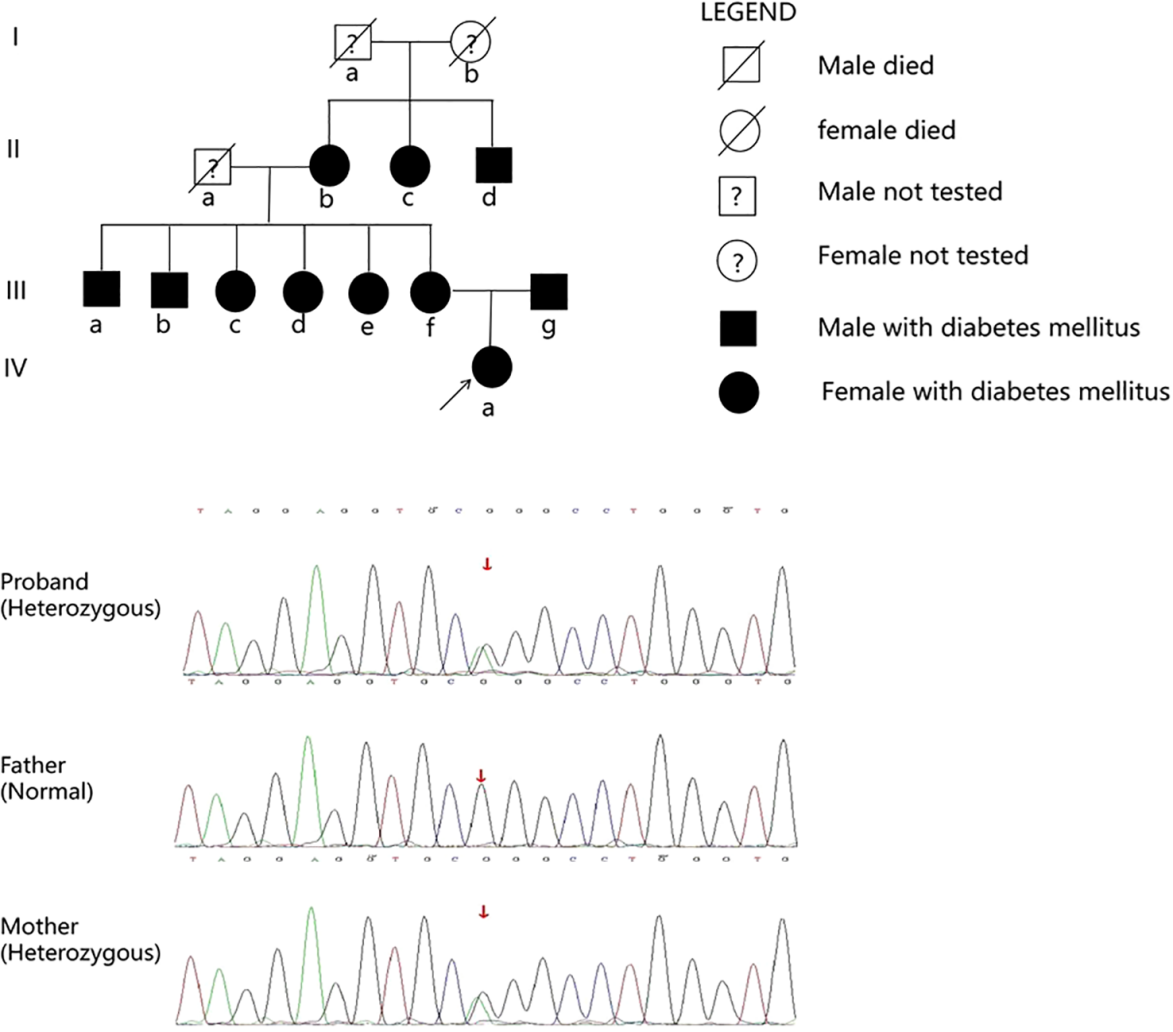
Family pedigree. IIIf and IVa carried the p.R301C mutation in the *KCNJ11* gene.

Whole exome sequencing was conducted to identify the causal gene in the proband. A heterozygous mutation of p.Arg301Cys (p.R301C, c.901C>T) was found in the KCNJ11 gene (accession number NM_000525.4, exon 1). The mutation was validated using Sanger DNA sequencing (**Figure 3**), and the same mutation was identified in her mother. No mutations in genes involved in the development of the pituitary were found. Written informed consent was obtained from the patient and her parents.

### Treatment

Based on these results, the patient was diagnosed with combined pituitary hormone deficiency (CPHD) caused by posterior pituitary ectopia and absent pituitary stalk. She was prescribed hydrocortisone, thyroid hormone, and estrogen, and scheduled for follow-up in our outpatient clinic. Considering the presence of GHD, which may be a major contributing factor to cirrhosis, recombinant human growth hormone (rhGH) was prescribed. Additionally, she was given IDegAsp (70% insulin degludec and 30% insulin aspart, 20 IU once before dinner), metformin (500mg twice a day), and canagliflozin (100mg once a day) to manage her glucose levels.

### Ethics statement

Written informed consent was obtained from the patient and her parents included in the study. Publication of a specific case report does not need ethical review in our institution.

## Discussion

Several studies have explored the connection between PSIS and nonalcoholic fatty liver disease (NAFLD). It has been reported that NAFLD and cirrhosis were diagnosed after the diagnosis of hypothalamic/pituitary dysfunction, with an average time of only 6.9 years from liver dysfunction to decompensated cirrhosis (2). Actually, the progression from NAFLD to cirrhosis typically takes 30 years (3). The prevalence of NAFLD in hypopituitary patients with GHD was found to be 77% in a Japanese study (4) and 54% in a Chinese study (5). It is important to note that untreated GHD, rather than other pituitary hormone deficiencies, plays a significant role in the development and fibrosis of NAFLD. Liver steatosis and elevated liver enzymes were observed to improve only after GH replacement therapy, but did not respond to glucocorticoid and thyroid hormone replacement (6). A retrospective study from Japan showed a significant reduction in serum liver enzyme levels and fibrotic markers in patients receiving GH (7). However, this finding was not replicated in another study from England, possibly due to differences in sample size, treatment duration, and GH dosage (8). It’s reported that GH and IGF1 could enhance whole body nutrient utilization and reduce inflammation, which would serve to ultimately improve insulin sensitivity and shift nutrients away from the liver. In addition, there are multiple liver-specific actions of GH and IGF that would favor prevention and protection against NAFLD progression (9). Thus, PSIS with GHD is the most likely cause of cirrhosis in our patient. Also, the role of hypothyroidism, hypoadrenocorticism, and hypogonadism in NAFLD could not be ruled out.In childhood, the most common manifestation of PSIS is growth retardation due to GHD. However, there have been reports of growth occurring without GH (10–12). This phenomenon was initially observed in patients with hypopituitarism after craniopharyngioma surgery, and the underlying mechanism remains unclear. Hyperinsulinism and elevated prolactin levels were observed in those patients with excessive growth (13). Since insulin is structurally similar to IGF-1, it may act as a growth stimulus partially through the IGF-1 receptor. Elevated prolactin levels may also normalize IGF-1 levels in GHD patients without hyperinsulinism (13). Leptin may also contribute to maintaining growth velocity by promoting the proliferation and maturation of chondrocytes (14). However, there have been cases where patients experienced normal growth without GH and had normal levels of insulin, prolactin, and leptin. In these patients, unknown growth-promoting factors may be present. Another study found that patients with CPHD had higher heights and SDS for height compared to patients with isolated GHD, possibly due to estrogen deficiency delaying epiphyseal fusion and prolonging growth (15). In addition, estrogens have a slightly antagonistic effect on the bioactivity of GH. A very low GH secretion would operate a greater effect than expected in the absence of estrogens (16). Our patient was not obese, and her insulin and prolactin levels were normal.

Genetic screening revealed a *KCNJ11* gene mutation (p.R301C) encoding ATP-sensitive potassium (K_ATP_) channel subunits. Previous studies have shown that this mutation reduces K_ATP_ channel expression on cell surfaces, causing rapid and spontaneous current decay and a gating defect called inactivation, contributing to the hyperinsulinism phenotype (17). All patients with the p.R301C mutation in the *KCNJ11* gene have been reported to exhibit CHI and hypoglycemia (17–19). However, there have been reports of other inactivating *KCNJ11* mutations leading to diabetes (19–21). Transgenic mice with inactivating K_ATP_ channels exhibit neonatal hypoglycemia with hyperinsulinemia, and adult mice show hyperglycemia with hypoinsulinemia and reduced β-cell numbers. Additionally, adult transgenic mice have elevated basal calcium levels and increased β-cell apoptosis (22). Compared to other mutations at this site (arginine 301 to glycine, histidine, or proline), the p.R301C mutation in *KCNJ11* only mildly reduces surface expression (18). We speculate that the patient and her mother transitioned from hyperinsulinemia to hyperglycemia, eventually developing diabetes due to age-related decline in β-cell function. The patient’s early hyperinsulinemia may have contributed to her tall stature and liver steatosis. To the best of our knowledge, this is the first case of diabetes mellitus caused by the p.R301C mutation in the *KCNJ11* gene.

Although the patient’s mother carried the same inactivating *KCNJ11* mutation and had diabetes, it is unknown if she had abnormal glucose levels during pregnancy. Breech delivery occurred without a clear reason, and mechanical rupture of the pituitary stalk led to panhypopituitarism and CPHD. In this patient’s case, early CHI caused by the inactivating *KCNJ11* mutation, along with delayed epiphyseal closure due to estrogen deficiency, may have contributed to her unusually tall height. If insulin and low GH levels were present, they would have continued to stimulate her linear growth. Additionally, untreated GH deficiency increased her visceral fat, leading to NAFLD and cirrhosis, ultimately requiring a liver transplant. NAFLD may have also contributed to the development of diabetes.

Biochemical evaluation of the patient’s thyroid function showed positive thyroglobulin antibody combined with high TSH and low FT4, suggestive of Hashimoto thyroiditis with subsequent primary hypothyroidism. However, a slightly elevated TSH level did not match the significantly reduced FT4 level. Insufficient compensatory pituitary secretion of TSH may be a probable interpretation for this.There were several limitations in this report. Firstly, the patient did not undergo a euglycemic clamp test for technical reasons. Secondly, because GH-releasing hormone, thyrotropin-releasing hormone and corticotrophin-releasing hormone were unavailable in China, we were unable to conduct relevant diagnostic stimulation tests. Lastly, we did not obtain genetic information or perform endocrine assessments on all family members.

## Conclusion

We present a unique case of PSIS with tall stature, liver cirrhosis, and diabetes. Genetic screening identified an inactivating mutation in the *KCNJ11* gene, which is known to be associated with CHI. We hypothesize that the combination of early hyperinsulinism caused by the mutation and delayed epiphyseal closure due to estrogen deficiency may have contributed to the patient’s remarkably tall height. The untreated GHD likely played a role in increasing visceral fat and leading to NAFLD and cirrhosis. Additionally, the decline in β-cell function with age and the presence of NAFLD might have contributed to the development of diabetes.

## Patient perspective

I had a challenging and potentially life-threatening birth experience. During my teenage years, while my female classmates began developing breasts and experiencing menstruation, I did not exhibit any signs of secondary sex characteristics. I sought medical help, but unfortunately, a definitive diagnosis was not reached. Another aspect that set me apart was my height, as I continued growing until my twenties. Standing at 186cm, I am taller than most women in China. Though I sometimes feel insecure about it, I have come to accept this reality.

At the age of 24, I received a diagnosis of diabetes. The doctor believed it could be attributed to my family history, as both my parents have diabetes. Consequently, I started taking oral hypoglycemic medication. Two years ago, a routine medical exam revealed that I had developed liver cirrhosis. Lacking sufficient knowledge about the condition, I regrettably did not seek medical treatment, leading to a recent episode of melena or gastrointestinal bleeding.

Before meeting Dr. Liu, I often felt like an unfortunate outcast who was different from others. However, Dr. Liu swiftly recognized my clinical symptoms and identified a potential link to PSIS. She explained the results of my genetic testing, which revealed that this gene mutation may have been responsible for all my conditions. While I cannot change my genes, most of my conditions can be controlled through proactive treatment.

I hope that my experience can offer support to other individuals diagnosed with PSIS. If promptly treated, I could have developed secondary sexual characteristics like my peers and potentially managed my height, preventing the occurrence of liver cirrhosis.

## Data availability statement

The data presented in this study will be made available by the authors upon request, without undue reservation.

## Ethics statement

The requirement of ethical approval was waived by the ethics committee of Tsinghua Changgung Hospital. The studies were conducted in accordance with the local legislation and institutional requirements. The participants provided their written informed consent to participate in this study. Written informed consent was obtained from the individual(s) for the publication of any potentially identifiable images or data included in this article.

## Author contributions

ZL: Conceptualization, Investigation, Writing – original draft. WZ: Supervision, Writing – review & editing. CC: Writing – review & editing. YW: Writing – review & editing. LX: Writing – review & editing. XW: Writing – review & editing. CJ: Writing – review & editing. JX: Conceptualization, Supervision, Validation, Writing – review & editing.
